# High-Throughput Yield Prediction of Diallele Crossed Sugar Beet in a Breeding Field Using UAV-Derived Growth Dynamics

**DOI:** 10.34133/plantphenomics.0209

**Published:** 2024-07-29

**Authors:** Kazunori Taguchi, Wei Guo, James Burridge, Atsushi Ito, Njane Stephen Njehia, Hiroaki Matsuhira, Yasuhiro Usui, Masayuki Hirafuji

**Affiliations:** ^1^ National Agriculture and Food Research Organization, Hokkaido Agricultural Research Center, Memuro Research Station, 9-4 Shinseiminami, Memuro, Kasai, Hokkaido 082-0081, Japan.; ^2^ National Agriculture and Food Research Organization, Central Region Agricultural Research Center, 3-1-3 Kannondai, Tsukuba, Ibaraki 305-8604, Japan.; ^3^ Graduate School of Agricultural and Life Sciences, The University of Tokyo, Nishi-Tokyo city, Tokyo 188-0002, Japan.

## Abstract

Data-driven techniques could be used to enhance decision-making capacity of breeders and farmers. We used an RGB camera on an unmanned aerial vehicle (UAV) to collect time series data on sugar beet canopy coverage (CC) and canopy height (CH) from small-plot breeding fields including 20 genotypes per season over 3 seasons. Digital orthomosaic and digital surface models were created from each flight and were converted to individual plot-level data. Plot-level data including CC and CH were calculated on a per-plot basis. A multiple regression model was fitted, which predicts root weight (RW) (*r* = 0.89, 0.89, and 0.92 in the 3 seasons, respectively) and sugar content (SC) (*r* = 0.79, 0.83, and 0.77 in the 3 seasons, respectively) using individual time point CC and CH data. Individual CC and CH values in late June tended to be strong predictors of RW and SC, suggesting that early season growth is critical for obtaining high RW and SC. Coefficient of parentage was not a strong factor influencing SC. Integrals of CC and CH time series data were calculated for genetic analysis purposes since they are more stable over multiple growing seasons. Calculations of general combining ability and specific combining ability in F1 offspring demonstrate how growth curve quantification can be used in diallel cross analysis and yield prediction. Our simple yet robust solution demonstrates how state-of-the-art remote sensing tools and basic analysis methods can be applied to small-plot breeder fields for selection purpose.

## Introduction

Sugar beet (*Beta vulgaris* L.) is an important root crop for sugar production in temperate regions [1]. The global area of land cultivated with sugar beets was 4.44 million ha in 2020. In comparison to the maximum of 9.43 million ha reached in 1976, the overall trend in area cultivated has decreased over the last 45 years [2]. However, average sugar beet yield is increasing and its productivity almost doubled from 6 t/10a in the 2010s from 3 t/10a in the 1970s.

Sugar beet varieties are typically hybrids that take advantage of heterosis. Heterosis, or hybrid vigor, is the phenomenon by which nonself-pollination results in progeny with greater vigor. In other words, progeny of different genotypes, heterotic groups within a species, or crosses between species exhibit greater biomass, development speed, and fertility than their parents [3–5]. Sugar beet is one of the crops in which heterosis has been used to obtain high yield performance [1,6–8]. Sugar beet breeders exploit heterosis by crossing with cytoplasmic male-sterile (CMS) lines [9]. Diallel cross analysis is a genetic selection tool designed to estimate genetic and nongenetic effects, quantify hybrid performance, and identify superior parental genotypes [10,11]. This method can efficiently estimate that the combining ability of parents with fewer mating combinations is used for selecting parental combinations in various crops [12–14]. In maize, crossing experiments using conventional pedigree data estimated heterosis of yield by predicting the degree of kinship between parents of F1s [15]. Subsequently, the genetic distance can be estimated using molecular markers [16]. A highly negative correlation has been observed between the coefficient of parentage (F) and root weight (RW) in sugar beet [17]. However, even if F between parents is similar, there can still be large variation in yield, suggesting nonlinear genotypic response to environmental variation. For this reason, field trials must also be conducted, which require substantial investment of time, energy, and resources.

Sugar beet is managed as a nonflowering root vegetative crop. Leaf area and root yield increase as long as the plant does not flower and development is not hampered by pests or disease. Although the genetic aspects of sugar beet performance have not been elucidated in detail, the effect of environmental, management, and crop physiological factors on sugar beet yield has been studied for many years [18]. Sugar beet begins accumulating sugar in its root very early in its growth cycle [19]. For healthy and unstressed sugar beet crops, the total amount of dry root mass is proportional to the amount of radiation intercepted by the canopy during growth [20]. Additional nitrogen application drives leaf growth and sugar production up to a genotype and environmentally dependent threshold [21,22]. Any factor restricting the speed of leaf surface area expansion directly reduces final production, and identifying ideal leaf area dynamics could be useful for breeders and farmers. Leaf area controls the interception of radiation, and its expansion is particularly important until full leaf cover is reached. In sugar beet, a leaf area index (LAI) of approximately 3 is recommended for maximum light use efficiency [23].

Capturing crop growth dynamics, such as foliar volume, LAI, and crop growth rate (CGR), can be useful for breeding, particularly as they can quantify interactions across years and fields. Growth models, such as SURCOS and GreenLab [24,25], have a complex physiological basis, requiring many parameters, yet relying on empirical functions with no mechanistic basis to partition assimilates within the crop [26]. For these reasons, a simple and high-throughput method to quantify crop growth dynamics and estimate sugar beet yield is highly desirable.

High-throughput phenotyping using UAVs has been successfully implemented at single plant, plot, and field-level traits in many crops [27–30] as well as in breeding fields [31,32]. Various methodologies have been developed to predict below-ground yield from above-ground information for potato [33], cassava [34], and sweet potato [35] using site-specific growth information, UAVs, and sometimes machine learning. In field-grown sugar beet, both manual photogrammetry [36] and aerial LiDAR [37] have been used to estimate traits including above-ground biomass and height, but they did not attempt to predict sugar beet root yield or SC. Cao et al. [38] developed a UAV technique using multispectral images to capture growth dynamics and indicators of performance, which they used to improve management and estimate root fresh weight but not SC. Another approach similarly used multispectral UAV imagery to describe canopy structure and color but did not attempt to estimate yield or SC [39]. UAV-based normalized difference vegetation index (NDVI) estimates from single time points were used to estimate sugar production in variable fertilization and irrigation treatments using a single cultivar with *R*^2^ up to 0.96 in one season [40]. They were primarily interested in improving late season fertilizer recommendation and found that the date with the best prediction varied by environment. A yield prediction approach comparing the ability of various vegetation indices collected at different time points was used to conclude that early season metrics were better predictors of yield than late season measurements [41]. Wang et al. [42] compared the ability of several sensors, vegetation indices, their combinations, and machine learning techniques to estimate SC of 185 varieties. This approach identified good techniques (maximum *R*^2^ of 0.64), but since all data were acquired on a single day and single season, the best technique could be different in a different season.

Our goal was to develop a high-throughput method to predict sugar beet RW and SC in small-plot breeding fields. The first step was to precisely capture canopy coverage and height dynamics. The second step was to develop a model to predict sugar beet RW and SC. Developing a method to quantify the genetic–environmental interactions on RW and SC using time series data could accelerate development of superior cultivars.

## Materials and Methods

### Plant material

A total of 20 genotypes were used in the 3 seasons: 3 cultivars (Amahomare, Monohikari, and Hokkai-Mitsuboshi), 1 self-incompatible line (NK-377 mm-O), 5 inbred lines, and 11 F1 hybrids developed by the National Agriculture and Food Research Organization (NARO)-Hokkaido Agricultural Research Center (HARC) in Japan. The 5 inbred lines (NK-184 mm-O, NK-195BRmm-O, NK-235BRmm-O, NK-237BRmm-O, and NK-388 mm-O) were selected to represent diversity in the Japanese Sugar Beet DIVersity set (JSBDIV) [43]. Taguchi et al. [43] should be referred to for the genealogical information of each inbred line and the method for calculating F. These 5 inbred lines and male sterility maintainers were selected for determining the yield combining ability and were self-fertilized for more than 6 generations to increase the degree of genetic fixation. In addition, CMS lines from these maintainers were backcrossed to CMS parents more than 5 times in succession, making them almost identical. They were efficiently crossed as the seed and pollen parents. The maintainer and CMS sets were represented as NK184, NK195BR, NK235BR, NK237BR, and NK388 and were used for actual breeding and as experimental materials. A total of 10 F1 accessions were obtained by half-diallel mating using these 5 parental genotype combinations.

### Evaluation of phenotypic traits

The accessions were evaluated in routine plant breeding trials (randomized block design) with 4 replicates at NARO-HARC, Japan, in 2018 and 2021 and 3 replicates in 2020. Field trials were conducted in NARO-HARC fields (42°53′28.3″N, 143°04′36.0″E, Memuro, Hokkaido Prefecture, Japan). The materials were seeded in paper pots on 2018 March 15, 2020 March 12, and 2021 March 11. Seedlings were transplanted into the field on 2018 April 26, 2020 April 20, and 2021 April 22. The individual plot size was 8.10 m^2^, and the final plant density was 60 plants per plot (~74,000 plants ha^–1^). In total, 240 plants (i.e., 60 plants/plot) were evenly distributed into 4 replications in 2018 and 2021. In 2020, 180 plants were evenly distributed into 3 replications of 60 plants/plot. Roots (52/subplot) were harvested by hand on 2018 October 17, 2020 October 6, and 2021 October 21. Yield components were investigated 175 days after transplanting (DAT) in 2018, 170 DAT in 2020, and 183 DAT in 2021. Fresh RW (RW, fresh RW/plant) and sucrose percentage of fresh weight (SC) were determined using the Hokunoshi Venema automated analysis system (Venema Automation B. V., Groningen, Netherlands) using the modified Sachs-Le Docte method [44]. Beet brei was clarified using Al_2_(SO_4_)_3_, and SC was determined using polarimetry. The data were averaged across replicates. Statistical analyses were performed using the statistical program package R 3.6.0 software.

Six genotypes were selected for additional manual phenotyping in 2018: NK195BR, NK235BR, NK388, and their first-generation hybrids (F1) NK235BR × NK195BR, NK195BR × NK388, and NK235BR × NK388. In the 4 replicates of these 6 genotypes, the number of leaves, plant length (PL) (length from base to the tip of the longest leaf per plant), and LAI using a photosynthetically active radiation measuring device (AccuPAR LP-80, currently METER Group Inc., Pullman, WA, USA) were measured every 7 to 10 days from June to September.

### Image acquisition from experimental fields

UAV flights were carried out in each field from approximately 1 week after transplanting. They were conducted 18 times in 2018, 13 times in 2020, and 20 times in 2021. A commercial-grade UAV (DJI Phantom 4 Pro, SZ DJI Technology Co. Ltd., Shenzhen, China) and an onboard camera (FC6310) with a resolution of 5,472 × 3,648 and a field of view of 84° with a focal length of 8.8 mm was used to acquire images with a flight height of 30 m for 2018 and 2020 and 15 m for the year 2021. Each mission required approximately 5-min flight time. Using the autonomous flight app Litch (VC Technology Ltd., London, UK), the flight mission was set to a double grid with nadir-only imagery for 2018 and 2020 and a single grid with both nadir and oblique imagery for 2021, with >80% overlap of the pictures with the front and sides. To obtain the precise geographical position for producing the georeferenced digital orthophoto map (DOM) and digital surface model (DSM) in the later structure from motion and multiview stereo (SfM-MVS) process, the ground control points were evenly distributed around the sites, and their geographic coordinates were measured using a real-time kinematic GPS.

**Fig. 1.**
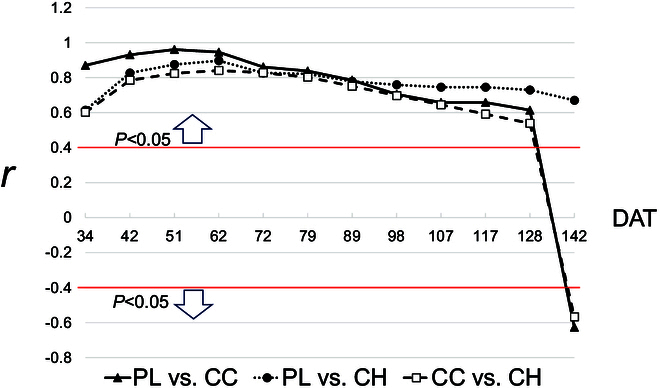
Plot of correlation coefficient among PL, CC, and CH values by observation day, 2018 data shown.

After flight and image collection, we followed a freely available, semi-automated UAV data processing pipeline (Fig. S1) [45]. First, we used the commercial SfM-MVS software “Pix4Dmapper Pro” (Pix4D SA, Lausanne, Switzerland) to reconstruct the experimental field in 3-dimensional (3D) and produce DOM and DSM. In the initial processing procedure, the “calibration method” was set to “alternative” and “camera optimization” was set to “all prior” to avoid reconstruction error in the flat and homogeneous agriculture field [46]. Second, we prepared shapefiles that can be used in the QGIS geographic information system (QGIS Development Team, https://www.qgis.org) using our previously developed preprocessing algorithm EasyMPE [47]. EasyMPE automatically detects the rows and columns of the field and generates a polygon with a predefined plot name/number based on field design. From the shapefile, the plots from both DOM and DSM can be extracted and saved as individual image files using our previously developed preprocessing algorithm, EasyIDP [48]. Individual plot images were segmented into vegetation and background using our previously developed machine learning algorithm easyPCC [49], and the sampling region was defined using random selection (Fig. S2). Subsequently, CC was calculated as the portion of vegetation area per unit soil area, and the CH was calculated as the difference between the CH (95th percentile of the plot DSM) and the ground height (5th percentile of the plot DSM). The design of the field experiment makes soil on either end of the target plot visible (Fig. S1F). The UAV-derived plant height value was validated using ground-truth data for PL from the 24 sample plots collected from June to September 2018. Pearson’s correlation analysis was performed between PL and CC and CH estimated values from the fitted curve for each investigation date (Fig. 1).

The estimated value was validated using ground-truth data for PL from the 24 sample plots collected from June to September 2018. Pearson’s correlation analysis was performed between PL and CC and CH estimated values from the fitted curve at each ground-truth measurement date.

Next, generalized linear model (glm) and nonlinear least squares (nls) functions were applied to the time series data of CC and CH using the R statistical software (R.project.org). The fitted constant terms, *a* and *b*, were calculated from the values of the actual measurements taken in the time series. Prediction error for each fitted growth curve was quantified using Akaike’s information criterion (AIC).

CC data were fit with the logit model using the following equation:

$$CC\left(x\right)=\frac{e^{\left(a+bx\right)}}{1+e^{\left(a+bx\right)}}$$

CH data were fit with the Gompertz model using the following equation:

$$CH\left(x\right)=ab^{e^{-cx}}$$

From the fitted growth curve of each plot, the integral value for each accession was calculated as CCintDAT and CHintDAT up to the specified number of DAT.

### Statistical analysis, diallel cross analysis and yield prediction

Hybrid crosses were performed following a half-diallel mating design (Method 2, Model 1 with fixed effects) according to Griffing [10]. Diallel analysis was conducted using the “DialleleAnalysisR” [50] package downloaded from the R main repository (https://r-project.org/), and the codes were based on the guidance of the package, in accordance with the implemented mating design. For each trait measured, the mean and range were calculated for the parental (*n* = 5) and F1s (*n* = 10) groups. All analyses were carried out using R. The statistical model is expressed as follows:

$$Y_{\mathrm{ij}}=m+g_{i}+g_{j}+s_{\mathrm{ij}}+e_{\mathrm{ij}}$$

where *Y_ij_* is the mean of the hybrid between “*i*” and “*j*,” *m* is the general mean, *g_i_* is the general combining ability (GCA) effect of “*i*,” *g_j_* is the GCA effect of “*j*,” *s_ij_* is the specific combining ability (SCA) effect of the hybrid between “*i*” and “*j*,” and *e_ij_* is the experimental error.

Analysis of variance (ANOVA) for each trait was performed to determine the influence of the estimated GCA and SCA, and genetic components estimated to be different from zero (*P* < 0.05) were considered to contribute significantly to the model. This analysis allowed the estimation of the genotypes and the reciprocal effect for all traits. The GCA of parents and SCA of individual combinations, along with the variance components, were estimated based using Griffing’s method 2 model 1. Griffing’s method 2 for diallel analysis considers the values for parents and one set of F1’s. Griffin’s model 1 uses a fixed effect model. The relative importance of GCA over SCA (GCA/SCA ratio) was estimated as the value of individual hybrids expressed as a ratio over the average of the trait.

Pairwise Pearson linear coefficients of correlation (*r*) were calculated, and the significance of correlations was evaluated using the Bonferroni test for RW, SC, and F, CC, and CH. Multiple regression analysis was performed with the RW and SC of each test plot as the dependent variable and F, CC, and CH as independent variables (Supplemental data).

## Results

### Yield and foliar growth of sugar beet in breeding fields

The temperature, precipitation, and sunshine duration of the 3 seasons are summarized in Table S1. In the 2018 season, it was hot and sunny from mid-April to early June. As a result, early season canopy growth progressed faster compared to the 2020 and 2021 seasons. In the 2020 season, there was drought from mid-May to mid-June, which likely contributed to reduced plant growth. In the 2021 season, precipitation was normal, temperatures were high, and sunshine hours were plentiful from late May to mid-June contributing to generally good plant growth.

A wide range of variation was observed for each trait in each year (mean ± standard deviation): for 2018, RW = 6.34 ± 1.20 (t/1,000 m^2^) (range: 3.48 to 8.10 t), SC = 17.49 ± 0.88% (range: 15.16 to 19.51%); for 2020, RW = 5.95 ± 1.12 (t/1,000 m^2^) (range: 3.46 to 7.78 t), SC = 15.01 ± 0.93% (range: 13.42 to 17.06%); for 2021, RW = 6.86 ± 1.20 (t/1,000 m^2^) (range: 3.97 to 8.49 t), SC = 15.54 ± 0.84% (range: 13.99 to 17.49%). ANOVA among accessions indicated significant differences (*P* < 0.001) in all trials (Table 1). Over the 3 seasons, both RW and SC were high in 2018, but in 2020, both RW and SC were low. In 2021, RW was the highest, but SC was low. Over the 3 years, the mean RW of the inbred lines was approximately 60% of the commercial cultivars. The mean RW of F1s was 80 to 90% of the commercial cultivars.

**Table 1.** Average values and ranges of variation of yield components in breeding field trials (2018, 2020, and 2021). Values followed by the same letter in a column within each variety are not significantly different at *P* < 0.05 [Tukey’s honestly significant difference (HSD) test]. ***P < 0.001.

Category	Varieties	F	2018		2020		2021	
			RW (t/10a)	SC (%)	RW (t/10a)	SC (%)	RW (t/10a)	SC (%)
Inbred line	NK-184mm-O	1	6.13 deg	15.16 a	5.30 cef	13.45 a	6.90 efh	14.90 acd
	NK-195BRmm-O	1	5.32 bcd	16.88 bc	4.99 cd	13.68 ab	5.67 bc	13.99 a
	NK-235BRmm-O	1	4.31 ab	18.24 fg	4.18 b	16.62 ij	4.80 ab	16.02 dfgh
	NK-237BRmm-O	1	3.55 a	17.39 bf	3.46 a	15.90 gh	3.97 a	16.63 gi
	NK-388mm-O	1	4.47 ac	16.54 b	4.79 bc	13.42 a	5.16 b	14.26 ab
F1s	NK195BRxNK184	0.19	6.86 fghi	17.10 bcd	5.97 fhi	14.62 cd	7.65g hjk	14.73 ac
	NK235BRxNK184	0.01	6.34 deg	18.28 fg	5.64 defg	16.09 hi	7.12 efgi	16.34 fgh
	NK237BRxNK184	0.03	6.74 fgh	17.52 bf	5.91 fhi	15.28 ef	7.54 fj	15.52 cf
	NK184xNK388	0.05	6.59 fgh	17.15 bce	7.01 jl	14.08 bc	7.98 ijk	15.15 bcde
	NK235BRxNK195BR	0.01	6.49 egh	18.04 defg	6.13 ghi	15.56 fh	6.68 df	15.66 cfg
	NK237BRxNK195BR	0	7.11 gj	18.03 defg	6.39 hj	15.67 fh	7.89 gjk	15.60 cf
	NK195BRxNK388	0.07	7.86 ij	16.80 bc	7.38 lm	13.86 ab	8.49 k	14.71 ac
	NK237BRxNK235BR	0.13	5.89 def	19.51 h	5.37 cef	17.06 j	6.32 cde	17.49 i
	NK235BRxNK388	0.03	6.53 fgh	18.22 efg	6.33 ghj	15.43 fg	6.84 dfg	16.21 efgh
	NK237BRxNK388	0.01	6.86 fghi	17.68 cf	6.62 ijk	14.84 de	7.36 fj	15.74 dfg
	NK377xNK388	0.38	6.94 ghi	16.81 bc	5.88 eh	14.52 cd	6.88 efh	14.93 acd
Self-imcompatible	NK-377mm-O	0.5	5.47 ce	16.55 b	5.19 ce	14.61 cd	5.85 bd	14.51 ab
Commercial variety	Monohikari	0	8.10 j	17.71 cf	7.55 lm	14.88 de	8.11 jk	16.03 efgh
	Amahomare	0	7.38 hj	18.83 gh	7.17 klm	15.77 fh	7.91 gjk	16.85 hi
	Hokkai_Mitsuboshi	0	7.87 ij	17.50 bf	7.78 m	14.85 de	8.17 jk	15.60 cf
*F* test (vs. varieties)			***	***	***	***	***	***
Mean in total			6.34	17.49	5.95	15.01	6.86	15.54
±SD			1.18	0.92	1.1	1	1.2	0.89
CV			6.19	2.39	3.87	1.17	4.99	2.42
LSD (5%)			0.56	0.59	0.38	0.29	0.49	0.53
LSD (1%)			0.74	0.79	0.51	0.39	0.65	0.71

LSD, Fisher’s least significant difference

As for plant growth, a significant difference in the number of leaves was observed from 34 DAT (*P* < 0.05). To take 3 genotypes in the 2018 season as an example, leaf number was 15, 14, and 13 for NK195BR × NK388, NK195BR × NK235BR, and NK235BR × NK388, respectively (Fig. S3). The rate of increase in the number of leaves per day was estimated using linear regression as approximately 0.53, 0.55, and 0.52 per day for NK195BR × NK235BR, NK235BR × NK388, and NK195BR × NK388, respectively. A significant difference in plant height was observed from 34 DAT (*P* < 0.001). Plant heights were approximately 29, 24, and 20 cm for NK195BR × NK235BR, NK235BR × NK388, and NK195BR × NK388, respectively. Plant height rankings of NK195BR × NK235BR, NK235BR × NK388, and NK195BR × NK388 did not change over the course of the season, and the maximum plant height at 116 DAT was 61, 53, and 51 cm, respectively. This indicates a connection between plant growth metrics (e.g. plant height and leaf number) with stable development from early in the season.

### Dynamic estimates of canopy growth in each experimental plot

**Fig. 2.**
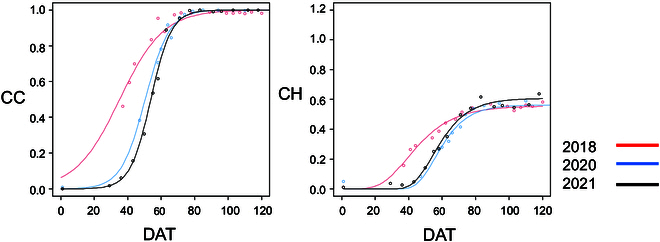
Comparison of the CC and CH growth pattern of the cultivar Monohikari in 3 seasons (2018, 2020, and 2021).

UAV flights were conducted every 30 days in each season and used to describe growth patterns. Manually measured PL and CH were highly correlated (Fig. 1). CC and CH adequately captured 3D canopy development. The CC growth pattern was fitted with a logistic model, and CH was fitted with the Gompertz model. The growth pattern of CC rapidly grew from 30 DAT and reached a plateau around 70 DAT in each season (Fig. 2). AIC values were calculated for several growing periods on a trial basis, and it was determined that the logistic model fit the CC data well. The growth pattern of CH tended to plateau slightly later, at approximately 80 DAT with minimal increases, dependent on genotype and environment, until 120 DAT (Fig. 1). Given this variation, the integral values of CC and CH plot models were calculated up to 120 DAT and used for genetic analysis. A wide range of variation was observed in both traits (mean ± standard deviation): CCint120 = 82.17 ± 6.36 (range: 65.75 to 90.56) and CHint120 = 37.82 ± 3.84 (range: 29.30 to 43.66) in 2018; CCint120 = 67.47 ± 3.96 (range: 57.34 to 72.95) and CHint120 = 28.90 ± 3.34 (range: 20.7 to 34.22) in 2020; CCint120 = 64.70 ± 3.93 (range: 54.12 to 69.45) and CHint120 = 31.80 ± 3.78 (range: 23.30 to 38.43) in 2021 (Table 2). ANOVA among accessions indicated significant differences (*P* < 0.001) in all trials. Similarly, ANOVA tests showed significant differences among accessions in every 30 DAT increment (*P* < 0.001), except CHint30 in 2020 and 2021 (Table S2).

**Table 2.** Average values and ranges of variation of dynamic traits in breeding field trials (2018, 2020, and 2021). Values followed by the same letter in a column within each variety are not significantly different at *P* < 0.05 (Tukey’s HSD test). ***P < 0.001.

Category	Varieties	2018		2020		2021	
		CCint120	CHint120	CCint120	CHint120	CCint120	CHint120
Inbred line	NK-184mm-O	84.11 df	30.72 ab	66.84 cd	24.65 ab	65.69 efh	26.07 b
	NK-195BRmm-O	71.25 b	29.30 a	59.81 ab	20.70 a	57.95 b	23.30 a
	NK-235BRmm-O	70.87 b	35.38 bcd	62.05 b	27.77 bd	58.03 b	28.31 bc
	NK-237BRmm-O	65.75 a	31.54 ab	57.34 a	24.86 ab	54.12 a	26.17 b
	NK-388mm-O	77.76 c	35.06 bc	69.26 df	27.98 bd	61.53 c	29.46 cd
F1s	NK195BRxNK184	86.37 efh	37.54 ce	67.23 cd	25.62 b	67.04 ghij	30.00 cd
	NK235BRxNK184	89.56 gh	39.12 cg	72.17 ef	30.94 cdf	69.16 jk	34.09 fgh
	NK237BRxNK184	90.56 h	41.68 eg	69.37 df	31.68 df	69.45 k	34.07 fgh
	NK184xNK388	88.12 fh	38.97 cg	72.95 f	33.01 ef	67.78 hik	34.47 fgh
	NK235BRxNK195BR	82.73 de	38.95 cg	66.63 cd	26.55 bc	65.33 efg	30.53 ce
	NK237BRxNK195BR	80.33 cd	40.32 eg	63.26 bc	26.97 bd	64.29 de	31.82 def
	NK195BRxNK388	87.47 fh	40.02 deg	70.07 df	29.02 bde	66.47 fi	32.99 eg
	NK237BRxNK235BR	82.98 de	40.82 eg	66.94 cd	31.71 df	65.14 efg	35.03 gh
	NK235BRxNK388	85.02 ef	43.05 fg	71.89 ef	32.87 ef	68.90 ik	36.79 hi
	NK237BRxNK388	85.22 ef	43.66 g	69.32 df	34.23 f	66.78 fi	38.43 i
	NK377xNK388	82.71 de	38.62 cef	69.58 df	30.56 cdf	64.81 ef	33.51 fgh
Self-imcompatible	NK-377mm-O	77.91 c	34.53 bc	67.06 cd	28.48 bde	62.56 cd	29.25 cd
Commercial variety	Monohikari	84.22 df	40.14 deg	69.56 df	32.82 ef	66.22 efh	35.78 hi
	Amahomare	85.40 efg	38.98 cg	68.52 de	28.37 bde	66.26 efh	32.93 eg
	Mitsuboshi	85.03 ef	38.03 ce	69.62 df	29.30 bde	66.51 fi	32.99 eg
*F* test (vs. varieties)		***	***	***	***	***	***
Mean in total		82.17	37.82	67.47	28.90	64.70	31.80
^±^SD		6.36	3.84	3.96	3.34	3.93	3.78
CV		1.96	4.84	1.96	5.35	1.25	3.26
LSD (5%)		2.28	2.59	2.19	2.56	1.15	1.47
LSD (1%)		3.04	3.45	2.93	3.43	1.53	1.96

### Genetic analysis for yield and dynamic traits

Diallel mating designs provided information about GCA and SCA among genotypes underlying the 4 target traits, namely, RW, SC, CCint120, and CHint120. All 4 traits showed significant GCA and SCA effects (*P* < 0.001), indicating that both additive and nonadditive gene actions controlled the expression of these traits (Table 3). These results were consistent with the significant *F* values from the ANOVAs when comparing all experimental accessions in the 3 seasons (Table 1). GCA/SCA ratios indicated the predominance of additive or nonadditive gene actions in determining a trait. SC 2020 and SC 2021 exhibited GCA/SCA > 1, suggesting that additive gene actions have a greater influence than nonadditive ones on the control of gene expression associated with these traits (Table 3). The remaining traits displayed higher SCA than GCA (GCA/SCA < 1), showing the predominance of nonadditive gene actions. These results indicate the quantitative variation in the genetic components of combining ability between the parents. Positive values indicate that the evaluated parent contributed alleles and genes that increased the mean of the trait.

**Table 3.** Mean squares of ANOVA in Griffing’s [10] half-diallel cross model. ***P < 0.001.

Year	Component	df	RW		SC		CCint120		CHint120	
			MS	Pr(>*F*)	MS	Pr(>*F*)	MS	Pr(>*F*)	MS	Pr(>*F*)
2018	GCA	4	0.93	***	2.30	***	62.54	***	11.34	***
	SCA	10	1.56	***	0.47	***	51.60	***	23.14	***
	Error	61	0.11		0.09		1.22		1.08	
2020	GCA	4	0.91	***	4.22	***	42.17	***	25.42	***
	SCA	10	1.18	***	0.20	***	12.67	***	10.12	***
	Error	42	0.14		0.03		0.65		1.05	
2021	GCA	4	1.58	***	2.50	***	23.59	***	19.64	***
	SCA	10	1.85	***	0.21	***	21.09	***	20.94	***
	Error	23	0.12		0.09		0.35		0.57	

The fixed effect of RW was higher in F1s crossed with NK195BR than in F1s crossed with NK184 (Table 4). The fixed effect of RW was highest in NK195BR × NK388, followed by NK237BR × NK388 and NK237BR × NK195BR. The fixed effect of SC was higher in F1s crossed with NK235BR than in F1s crossed with NK237BR, with NK237BR × NK235BR being the F1 with highest for SC. The fixed effect of CCint120 tended to be higher in F1s crossed with NK184 than with other parents, and NK237BR × NK184 was F1 with the highest fixed effect for CCint120. The fixed effect of CHint120 was higher in F1s crossed with NK237BR than in others, and NK237BR × NK388 and NK237BR × NK184 were the F1s with first and second highest values over the 3 seasons. Although the magnitude of the response differed depending on the year, CHint120 tended to be lower in F1s crossed with NK195BR than in other F1s (Tables 2 and 4). Table 5 shows a simple correlation matrix between traits in the diallel cross model. According to this matrix, F was consistently negatively correlated with RW, while CCint120 and CHint120 were consistently positively correlated with RW, with CCint120 being more highly correlated with RW. CCint120 and CHint120 were also correlated with each other.

**Table 4.** GCA/SCA ratio and fixed effect of RW, SC CCint120, and CHint120. Griffing’s [10] method 2 model 1 (fixed effects) of diallel analysis was used.

Genetic component	RW			SC			CCint120			CHint120		
	2018	2020	2021	2018	2020	2021	2018	2020	2021	2018	2020	2021
GCA	0.12	0.11	0.21	0.32	0.60	0.34	8.76	5.93	3.32	1.47	3.48	2.72
SCA	1.45	1.05	1.73	0.39	0.17	0.12	50.38	12.02	20.74	22.06	9.07	20.35
GCA/SCA	0.08	0.11	0.12	0.82	3.56	2.85	0.17	0.49	0.16	0.07	0.38	0.13
Fixed effect												
NK-184mm-O	0.34	0.14	0.60	−0.66	−0.46	−0.18	4.51	1.91	2.64	−1.10	−0.13	−0.72
NK-195BRmm-O	0.36	0.24	0.27	−0.18	−0.45	−0.63	−1.69	−2.18	−1.24	−1.58	−3.12	−2.40
NK-235BRmm-O	−0.36	−0.34	−0.52	0.79	1.02	0.67	−1.32	−0.05	−0.42	0.89	0.88	0.51
NK-237BRmm-O	−0.39	−0.42	−0.43	0.36	0.63	0.59	−2.95	−2.64	−1.95	0.44	0.41	0.52
NK-388mm-O	0.05	0.39	0.09	−0.30	−0.74	−0.44	1.44	2.96	0.97	1.34	1.95	2.10
NK195BRxNK184	0.09	−0.10	−0.04	0.44	0.50	0.07	1.67	0.49	0.92	2.48	0.30	1.96
NK235BRxNK184	0.29	0.15	0.33	0.65	0.50	0.34	4.49	3.30	2.49	1.58	1.61	2.54
NK237BRxNK184	0.72	0.50	0.91	0.32	0.07	−0.55	7.12	3.09	4.17	4.59	2.82	2.94
NK184xNK388	0.13	0.78	0.80	0.61	0.24	0.06	0.29	1.08	1.03	0.99	2.62	2.60
NK235BRxNK195BR	0.42	0.53	0.29	−0.07	−0.05	0.18	3.87	1.85	2.14	1.89	0.22	1.03
NK237BRxNK195BR	1.07	0.88	1.27	0.36	0.45	0.36	3.10	1.07	2.52	3.71	1.11	2.51
NK195BRxNK388	1.37	1.06	1.51	−0.22	0.02	0.17	5.85	2.28	2.35	2.51	1.62	2.48
NK237BRxNK235BR	0.58	0.43	0.66	0.86	0.37	0.57	5.37	2.62	2.88	1.74	1.84	2.04
NK235BRxNK388	0.77	0.58	0.53	0.23	0.11	0.50	3.02	1.98	3.81	3.08	1.46	2.72
NK237BRxNK388	1.13	0.95	0.95	0.11	−0.09	−0.14	4.85	1.99	3.29	4.14	3.29	4.78

**Table 5.** The correlation coefficient of matrix among traits in the diallele cross. ***P < 0.001, **P < 0.01.

Year	Trait	RW	SC	F	CC120int	CH120int
2018	RW	−	0.01	−0.80	0.81	0.61
df = 58	SC		–	−0.44	0.04	0.52
	F	***	***	–	−0.77	−0.82
	CC120int	***		***	–	0.65
	CH120int	***	**	***	***	–
2020	RW	–	−0.26	−0.79	0.69	0.50
df = 43	SC		–	−0.27	−0.18	0.26
	F	***		–	−0.61	−0.64
	CC120int	**		***	–	0.68
	CH120int	***		***	***	–
2021	RW	–	−0.19	−0.76	0.81	0.55
df = 54	SC		−	−0.29	0.03	0.38
	F	***		–	−0.77	−0.81
	CC120int	***		***	–	0.71
	CH120int	***	***	***	***	–

### Prediction of sugar beet RW and SC

**Fig. 3.**
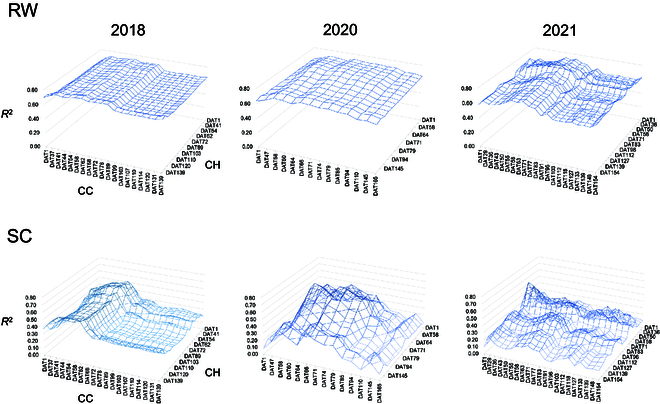
The multiple regression in the diallel cross (2018, 2020, and 2021). Coefficients of determination (*R*^2^) of RW (top) and SC (bottom) for each DAT combination of CC and CH.

**Fig. 4.**
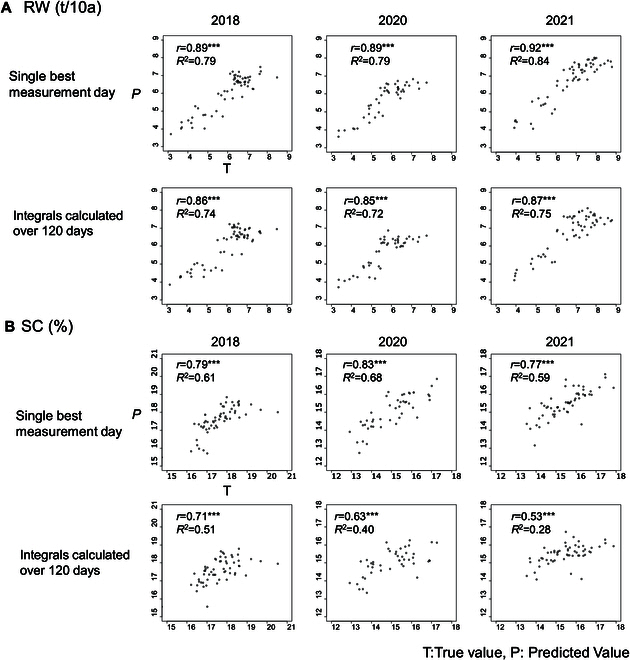
The multiple regression in the diallel cross (2018, 2020, and 2021). Correlation between actual RW and predicted RW (A) and SC actual and predicted SC (B) using the multiple regression model with measurements from selected individual time points and integrals calculated over 120 days. ***P < 0.001.

Multiple regression analysis was performed on RW and SC using 3 variables: F as X1, CC as X2, and CH as X3. Partial regression coefficients for RW in the 3 seasons were highly and consistently significant in F, CC, and CH (Table 6). The *R*^2^ between actual and predicted RW at a given DAT combination of CC and CH showed peaks around late June (from DAT60 to 75), although *R*^2^ for CH was the highest after DAT 130 in 2018 (Fig. 3). The highest correlation coefficients between actual and predicted RW using multiple regression were 0.89 for both CC-DAT62 and CH-DAT131 in 2018, 0.89 for CC-DAT71 and CH-DAT66 in 2020, and 0.91 for CC-DAT71 and CH-DAT63 in 2021 (Fig. 4 and Table 6).

**Table 6.** The partial correlation coefficient of multiple regression analysis for RW (upper panel) and SC (lower panel) with F (X1), CC (X2), and CH (X3) in the diallel cross. ***P < 0.001, **P < 0.01, and .*P* < 0.1.

Trait			2018				2020				2021	
RW	Variable	Estimate	*P*	*t* value	Variable	Estimate	*P*	*t* value	Variable	Estimate	*P*	*t* value
	Constant term	6.28	***	5.19	Constant term	4.52	***	5.43	Constant term	6.30	***	5.15
	F (X1)	-1.84	***	−6.33	F (X1)	−1.83	***	−9.09	F (X1)	−2.58	***	−7.96
	CC-DAT62 (X2)	3.99	***	4.83	CC-DAT71 (X2)	5.47	***	5.28	CC-DAT71 (X2)	7.08	***	7.61
	CH-DAT131 (X3)	−6.16	***	3.67	CH-DAT66 (X3)	−8.96	***	−3.69	CH-DAT63 (X3)	−14.94	***	−5.83
SC	Variable	Estimate	*P*	*t* value	Variable	Estimate	*P*	*t* value	Variable	Estimate	*P*	*t* value
	Constant term	17.70	***	11.68	Constant term	17.61	***	16.24	Constant term	10.76	***	9.39
	F (X1)	−0.69		−1.60	F (X1)	−0.81	**	−2.96	F (X1)	0.78	.	1.99
	CC-DAT62 (X2)	−6.97	***	−6.80	CC-DAT66 (X2)	−10.87	***	−8.54	CC-DAT29 (X2)	−136.94	***	−6.81
	CH-DAT58 (X3)	15.67	***	5.02	CH-DAT66 (X3)	18.54	***	6.21	CH-DAT58 (X3)	27.35	***	6.49

Partial regression coefficients for SC were highly significant for CC and CH in all 3 years and was significant for F in 2020 (Table 6). The *R*^2^ of correlation between actual and predicted SC at a given DAT combination of CC and CH showed peaks around late June (DAT 60) through late July (around DAT90) over 3 years (Fig. 3). The highest correlation coefficients between SC and predicted SC by multiple regression at CC-DAT62 and CH-DAT58 in 2018 was 0.79, CC-DAT66 and CH-DAT66 in 2020 was 0.83, and CC-DAT29 and CH-DAT58 in 2021 was 0.77 (Fig. 4 and Table 6).

**Fig. 5.**
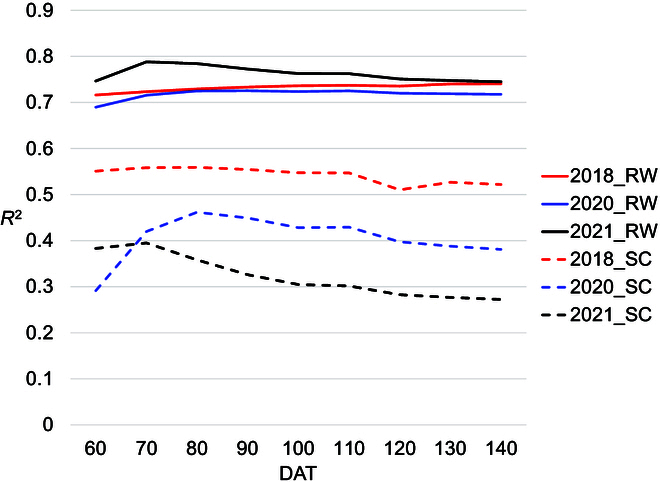
The multiple regression in the diallel cross (2018, 2020, and 2021). Coefficients of determination (*R*^2^) of RW and SC integrals calculated for each DAT combination of CC and CH.

CC and CH growth patterns of individual genotypes in the 3 trials are presented in Fig. S4. Integral values of every 10-day period from 60 to 140 DAT were calculated and used to predict RW and SC of all genotypes in each trial. Combinations of integral values taken at the same DAT are presented in a line plot in Fig. 5, and all combinations of CC and CH at different time periods are presented in Fig. 3. RW predictions at matched times tended to be uniformly high from 60 to 140 days, with *R*^2^ values between 0.7 and 0.8 (Fig. 5). Predictions of SC tended to be slightly higher early in the season (60 to 80 DAT), achieving *R*^2^ values between 0.29 and 0.56 (Fig. 5). CC and CH integral values from different measurement times achieved higher *R*^2^ values in the most challenging season than predictions at the same DAT (0.44 in 2021 using CCint70 and CHint60), but the best combination was different in different trials (Supplemental data2).

## Discussion

Environmental variation has a strong and difficult to predict impact on plant phenotypes. Therefore, selection in multiple environments is considered necessary by most plant breeders. The high cost of measuring yield from many small plots makes high-throughput, remote sensing-based selection criteria attractive. In this experiment, genetic and environmental effects on sugar beet growth patterns were captured using UAV-based CC and CH metrics (Fig. 2). CC captures horizontal canopy growth, while CH captures vertical growth, which is important following canopy closure. While strong associations were found between performance and CC or CH at specific measurement days, the impact of environmental and genotypic variation precludes the ability to recommend collecting data at only a single time point. Previous research achieved similar predictions of RW and higher prediction of SC using either one cultivar in multiple seasons or many cultivars in one season. We tested 20 genotypes in 3 years. Our integral approach co-optimizes the ability to predict RW and SC with both genotypic and environmental variation and, for this same reason, appears to have more limited prediction potential. The integrated values of CC and CH (CCint120 and CHint120) were used to quantify combining ability. This diallel analysis showed that SCA was larger than GCA and demonstrated that CC and CH traits can be useful criteria for genetic analysis. The diallel analysis further showed that SC had a higher GCA/SCA ratio than RW for 3 years in our study (Table 3). This was consistent with the results of previous reports, indicating a large dominant effect on RW and a large additive effect on SC [51]. A high correlation was observed between coefficient of parentages (F) and sugar yield in a controlled cross-experiment using a single pollen parent [17]. However, in the current set of more diverse genotypes, F did not show a high correlation with SC.

A relationship was found between the CC and CH growth patterns and RW and SC that were independent of individual mating combination. Correlation coefficients between individual CC- and CH-based predictions of RW and SC and actual RW were highest in late June (around DAT60) among 3 years of experiments (Fig. 3). In healthy and unstressed sugar beet, the total amount of accumulated dry matter is proportional to the amount of radiation intercepted by the canopy during growth [20]. In high latitude areas, such as Hokkaido, photosynthetically active radiation reduces rapidly from the summer solstice, which may help explain why relatively higher correlations between predicted and actual values were observed in late June. As additional evidence for the importance of early season growth, RW and root SC were higher in 2018, when favorable environmental conditions contributed to faster early growth than in the other 2 years. In contrast, a high correlation coefficient was also produced by CH 130 DAT in 2018. Kenter et al. [52] used an accurate but laborious approach to track dry matter accumulation of both leaves and taproot in many environments growing only a single cultivar at a time. They found higher temperatures early in the growth period and high solar radiation during the first 65 days after sowing and again in October favored root growth. Regardless of the mechanism, correlation analysis and multiple regression analysis of the data indicate that CC and CH metrics at the first inflection point of the growth curve (around 60 DAT) are strong predictors of RW and SC.

Interestingly, the CC coefficient in multiple regression analysis was positive for RW, while the CH component was negative for RW, but the opposite was observed for SC (Table 6). For both metrics, early season measures tended to be most powerful, except for CH in 2018. This may indicate that CC may be the primary driver of RW, while CH is the primary driver of SC. However, both are key indicators of genotypic and environmental variation and are likely both related to competitive ability and ability to accumulate RW and sugar. The F1 produced by NK195BR × NK388 had the highest RW among the diallel cross combinations and tended to have a large CC and a small CH, suggesting that CC may be more important than CH for RW. This point, as well as the previous points about the importance of early canopy growth, need to be examined in detail in the future, as vigorous early vegetative growth may be a breeding target [41].

Long-term studies highlight how drought and soil nitrogen availability, pests, and disease can limit sugar beet productivity ([53–55]). While these limitations were not present in the current analysis, frequent UAV data collection flights and the use of CC and CH integrals could help build predictive power in challenging environments with more variable and complex growth patterns at the level of hundreds of plots per hour with high precisions.

The high cost of measuring yield from many small plots makes high-throughput, remote sensing-based selection criteria attractive for breeding. The open-source tool used in our method to process and divide UAV data into user-defined field plots and calculate height, volume, and coverage is available online (http://cse.naro.affrc.go.jp/aitoh/PREPs/). Our data demonstrate that it is possible to achieve high selection efficiency by using nonconventional selection criteria, such as growth curves to predict RW and root SC. Furthermore, using remote sensing to quantify canopy growth dynamics has utility for parameterizing simulation models for specific agro-climatic regions. Machine learning-based approaches, in accord with the Breeding 4.0 concept [56], may offer additional tools to increase prediction accuracy and utilize genomic information. This study presents a method to use UAV time series data to predict RW and SC in a nonflowering root crop, including a range of cultivars and breeding accessions in 3 different seasons. Application of this approach to other field crops, particularly vegetatively harvested root crops, may produce similarly useful insights.

## Conclusion

We developed and demonstrated a UAV-based, high-throughput and easy to implement technique to use crop growth dynamics to estimate root weight and sugar yield. The simple and robust approach can be used to enhance breeder decision-making by providing a pre-harvest selection criterion for all accessions in the field, thus reducing the needs for laborious manual measurement. The UAV approach could also be extended to guide precision fertilization in production fields.

## Data Availability

The original contributions presented in the study are included in the article/Supplementary Materials. Further inquiries can be directed to the corresponding author.

## Supplementary Materials

Supplementary 1 Tables S1 and S2 Figs. S1 to S4 Supplemental data Supplemental data2
