# Supplementary material for: High-Throughput Yield Prediction of Diallele Crossed Sugar Beet in a Breeding Field Using UAV-Derived Growth Dynamics
Source: Plant Phenomics. 2024 Jul 29;6:0209. doi: 10.34133/plantphenomics.0209 (PMC11283879; doi:10.34133/plantphenomics.0209)
Supplement: Supplementary 1 — Tables S1 and S2 Figs. S1 to S4 Supplemental data Supplemental data2 [file plantphenomics.0209.f1.zip › supplemental Figures.pdf]

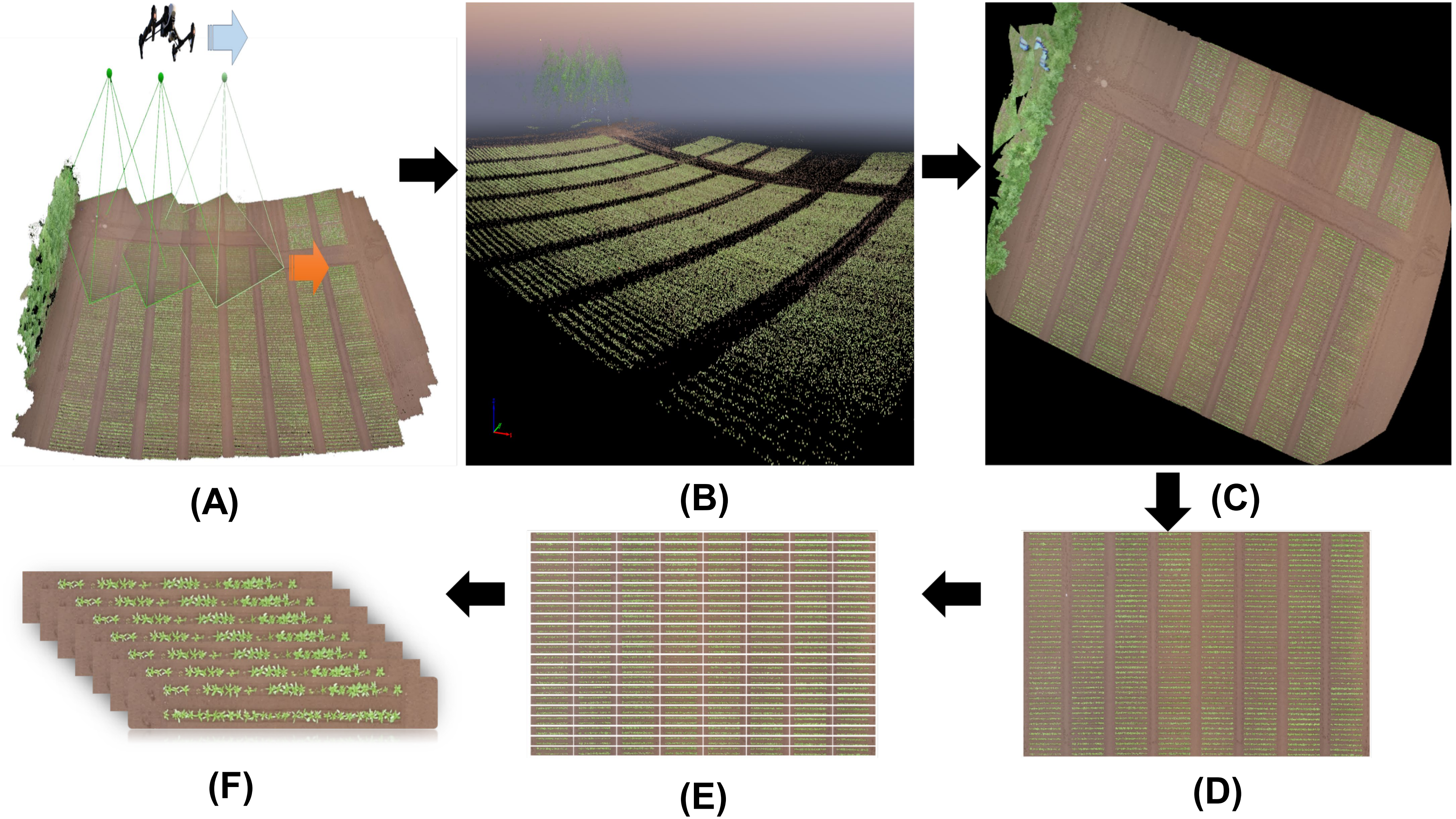

Supplemental Figure 1. UAV data processing pipeline,
(A) UAV flight with > 80% overlap of image acquisition, (B) 3D point cloud generated from UAV images using SfM-MVS software "Pix4Dmapper Pro," (C) DOM generated from 3D point cloud, (D) experiment region cropping and rotation, (E) plot segmentation based using on shape file generated based on experiment design, (F) cut individual plots images.

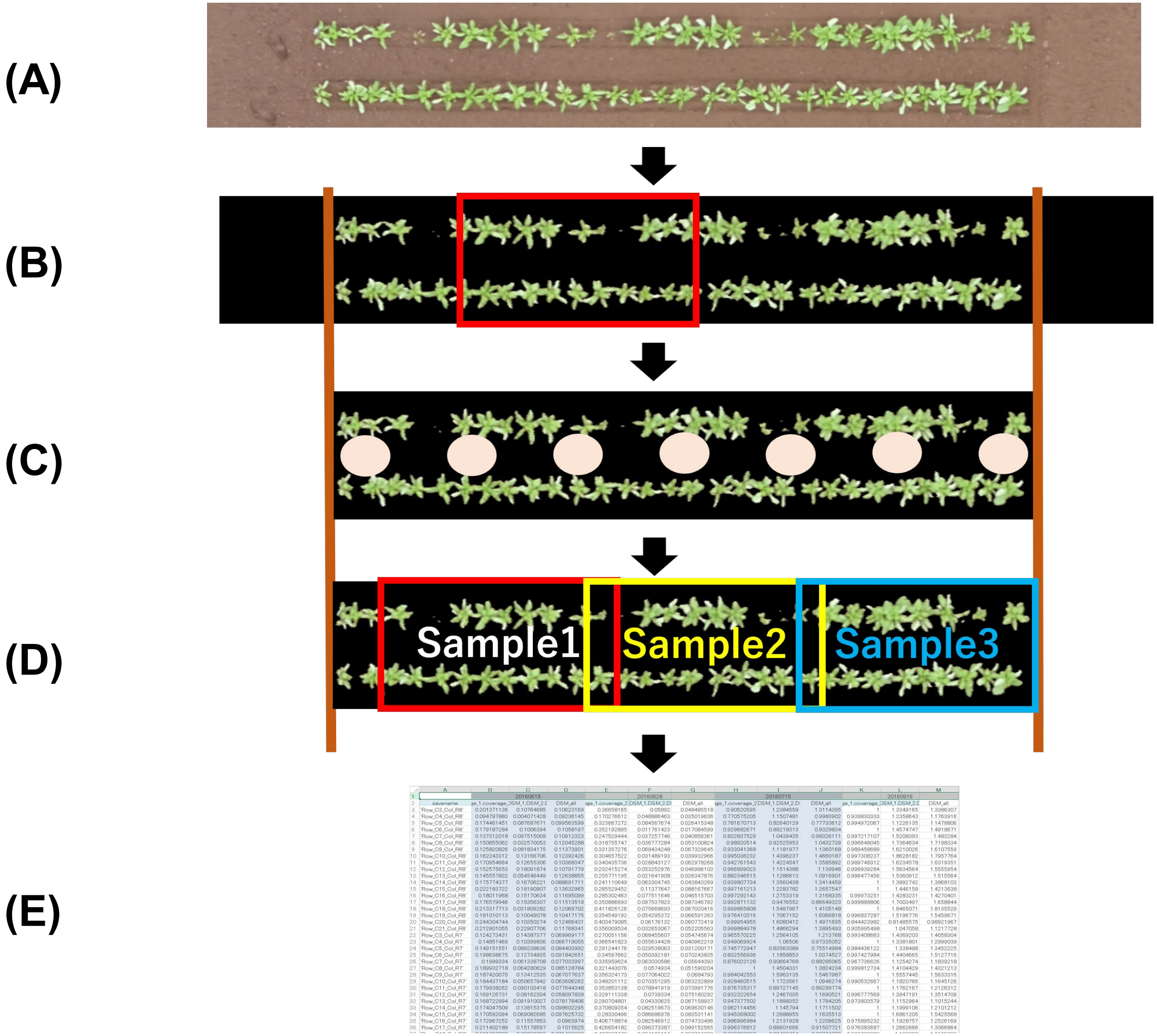

Supplemental Figure 2. Process of trait extraction from plot image: (A) original plot image, (B) plot image after the binarization process, (C) sampling region determination (dots indicate the seeds location of each sampling region), (D) randomly selected sampling region, and (E) extracted traits in csv format.

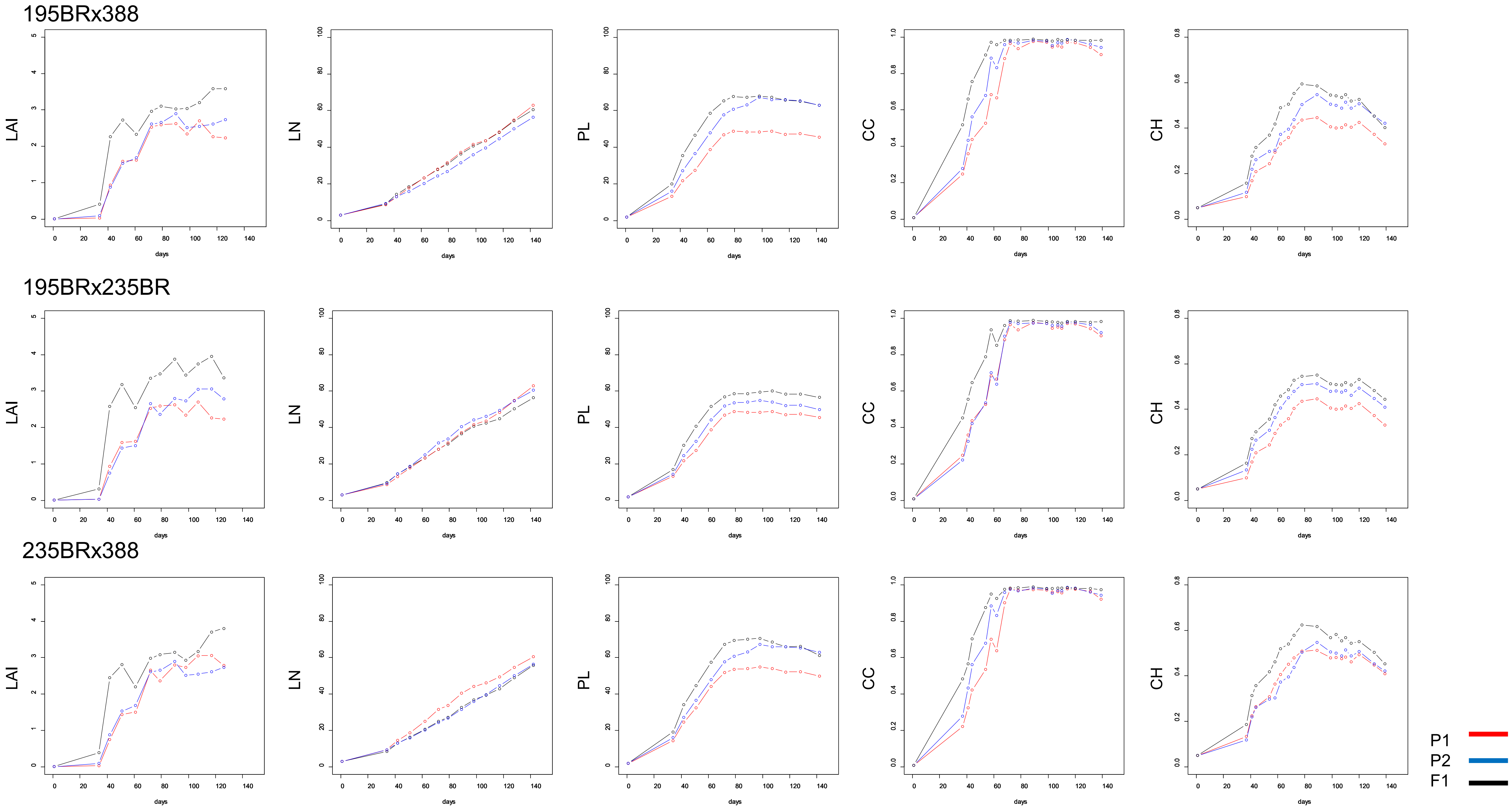

Supplemental Figure 3. Comparing the actual value of LAI, LN, PL, CC, and CH of three F1s and their parental lines (2018).

Supplemental Figure 4. The CC and CH growth pattern of each variety

CC (2018)

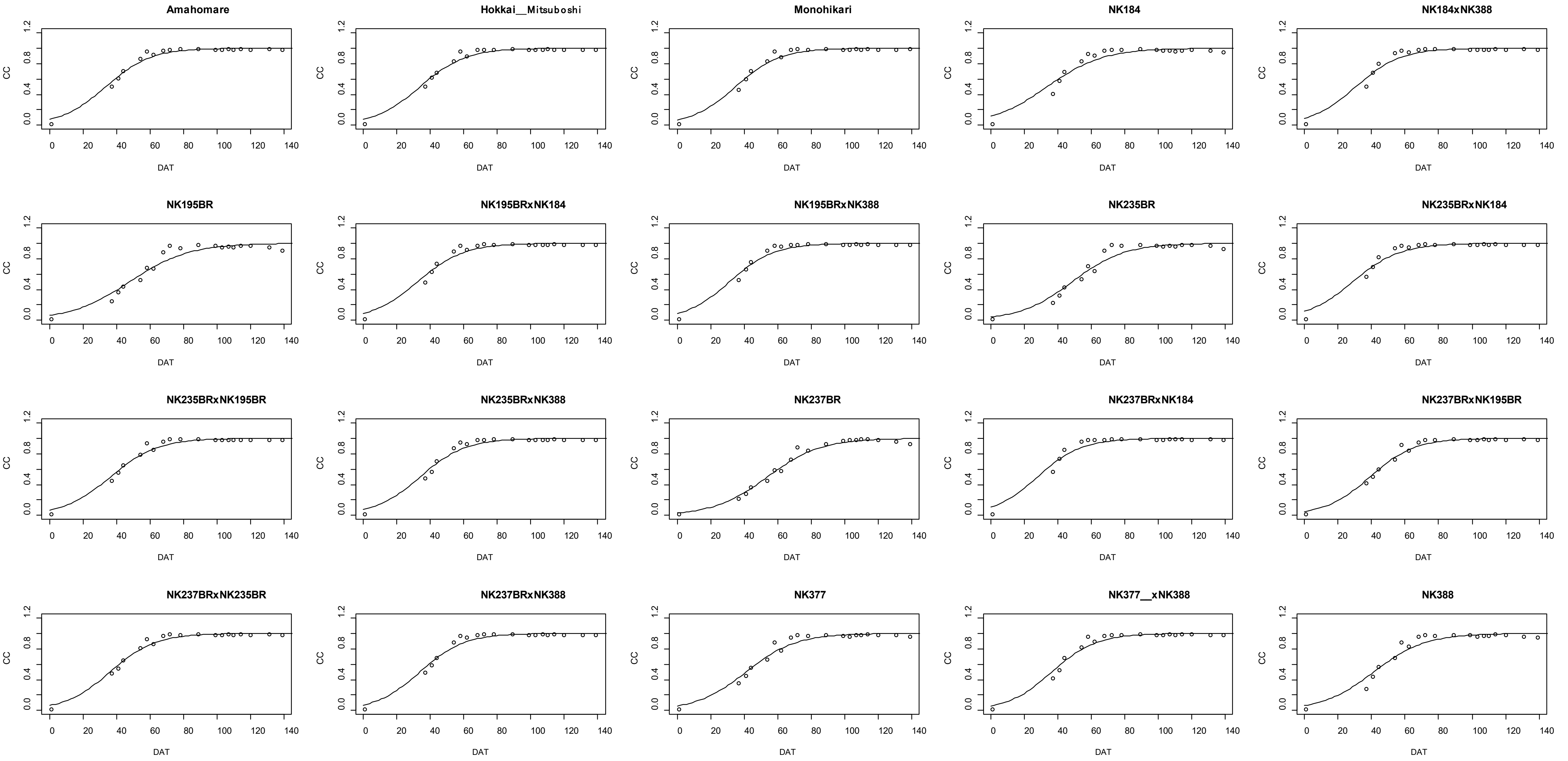

CC (2020)

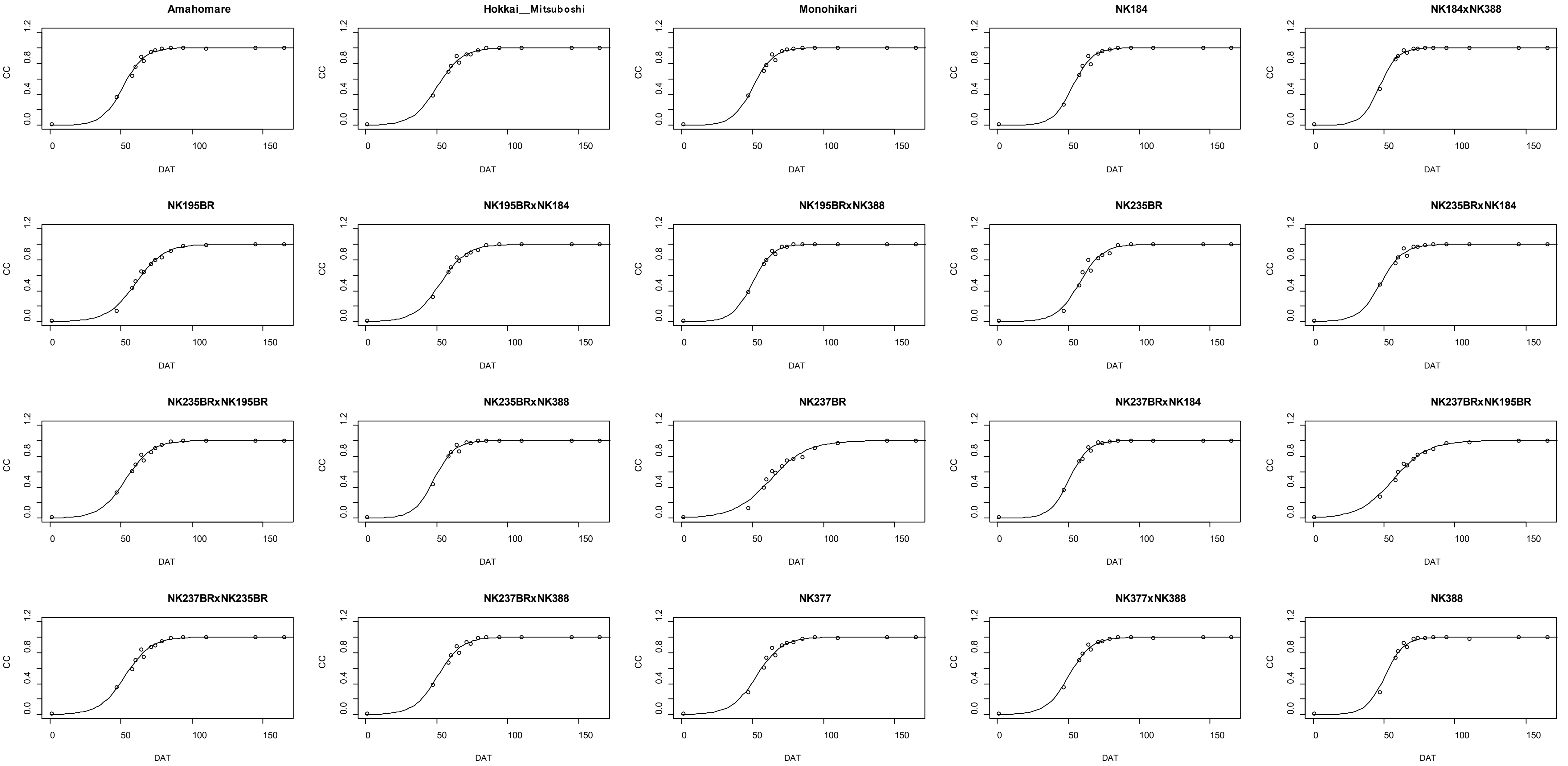
Amahomare
Hokkai__Mitsuboshi
Monohikari
NK184
NK184xNK388
NK195BR
NK195BRxNK184
NK195BRxNK388
NK235BR
NK235BRxNK184
NK235BRxNK195BR
NK235BRxNK388
NK237BR
NK237BRxNK184
NK237BRxNK195BR
NK237BRxNK235BR
NK237BRxNK388
NK377
NK377xNK388
NK388
CC
DAT
0.0 0.4 0.8 1.2
0 50 100 150

CC (2021)

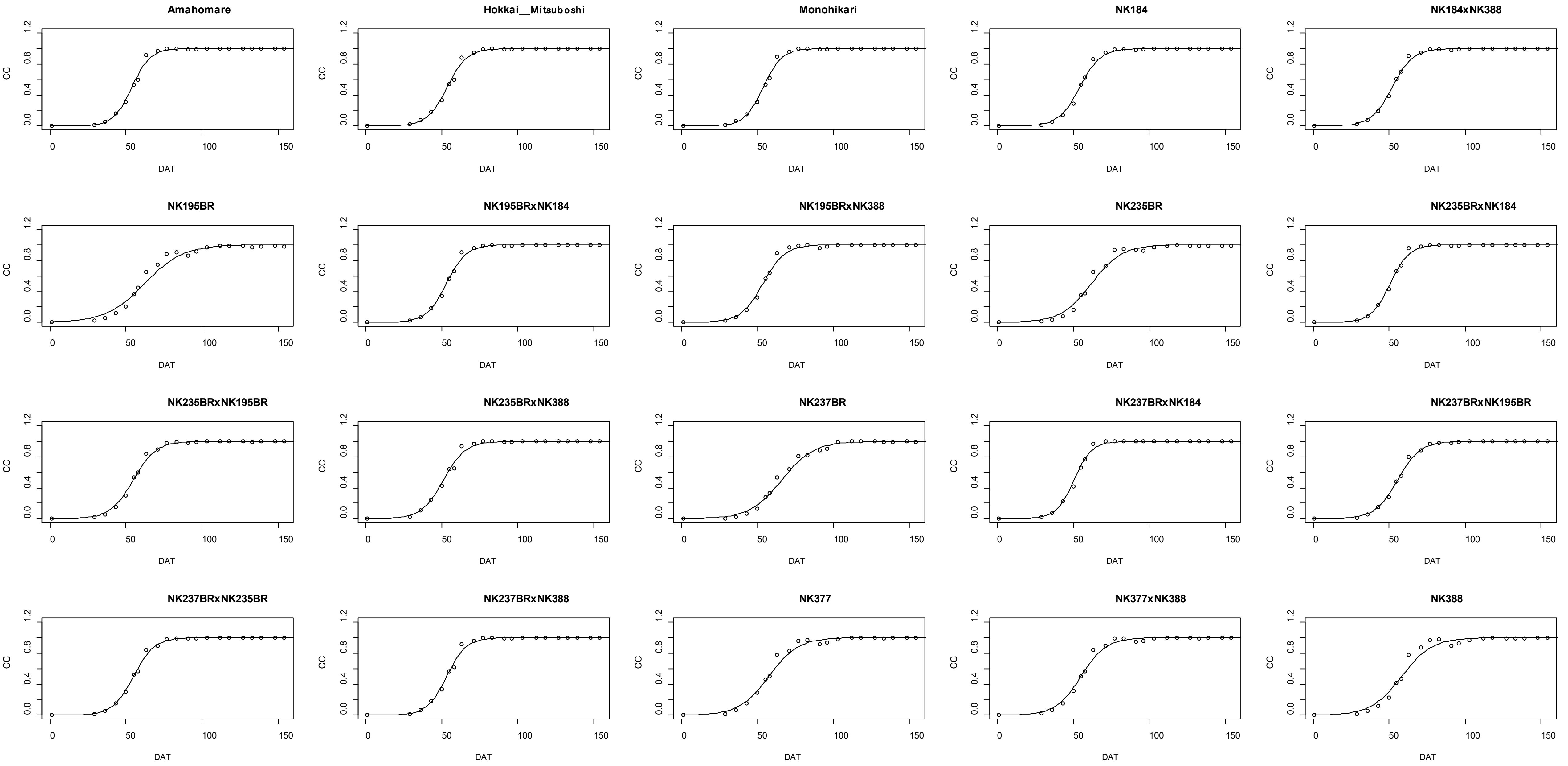
Amahomare
Hokkai__Mitsuboshi
Monohikari
NK184
NK184xNK388
NK195BR
NK195BRxNK184
NK195BRxNK388
NK235BR
NK235BRxNK184
NK235BRxNK195BR
NK235BRxNK388
NK237BR
NK237BRxNK184
NK237BRxNK195BR
NK237BRxNK235BR
NK237BRxNK388
NK377
NK377xNK388
NK388
CC
0.0
0.4
0.8
1.2
DAT
0
50
100
150

CH(2018)

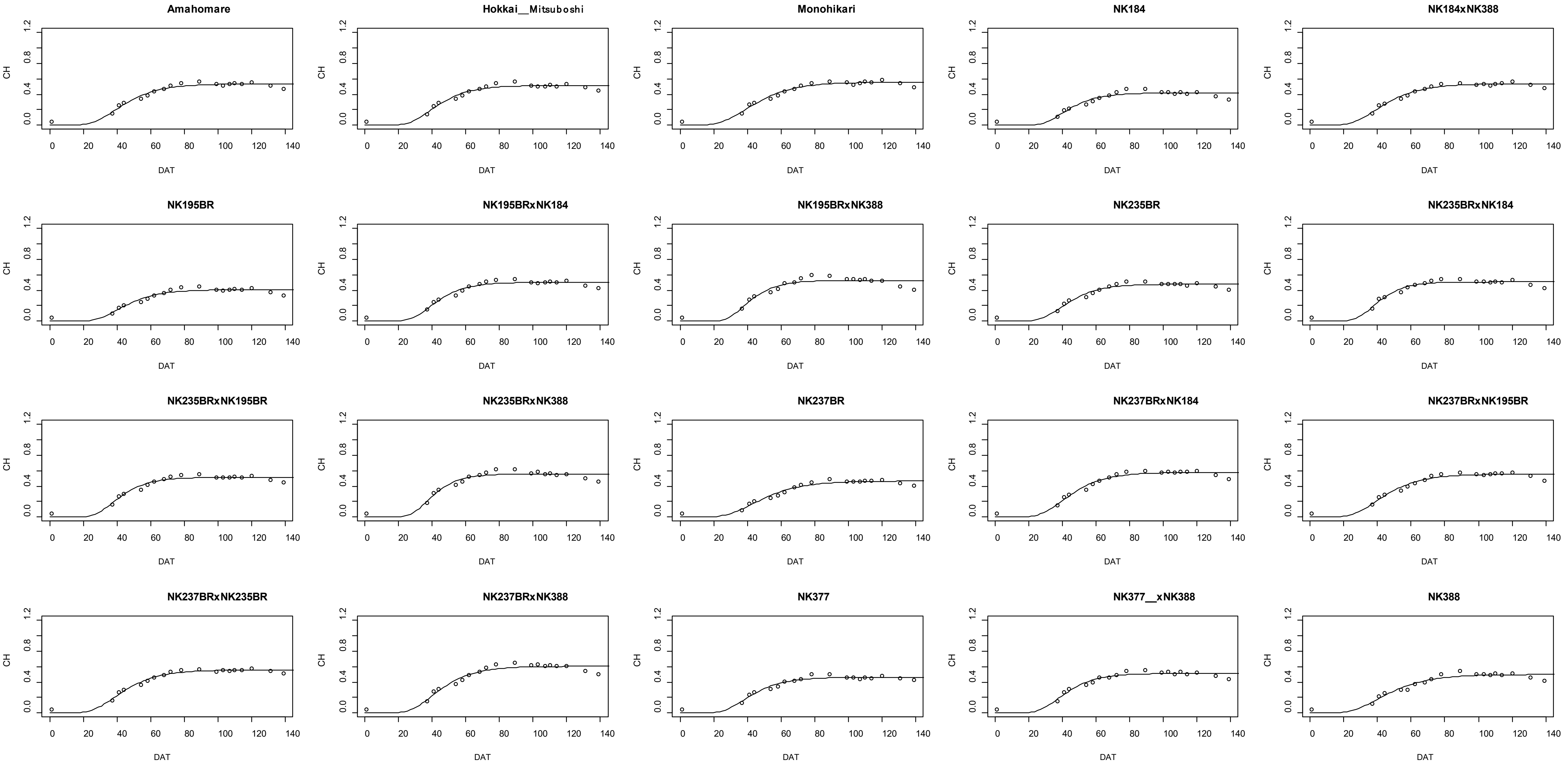
Amahomare
Hokkai__Mitsuboshi
Monohikari
NK184
NK184xNK388
NK195BR
NK195BRxNK184
NK195BRxNK388
NK235BR
NK235BRxNK184
NK235BRxNK195BR
NK235BRxNK388
NK237BR
NK237BRxNK184
NK237BRxNK195BR
NK237BRxNK235BR
NK237BRxNK388
NK377
NK377__xNK388
NK388
CH
DAT
0.0
0.4
0.8
1.2
0
20
40
60
80
100
120
140

CH (2020)

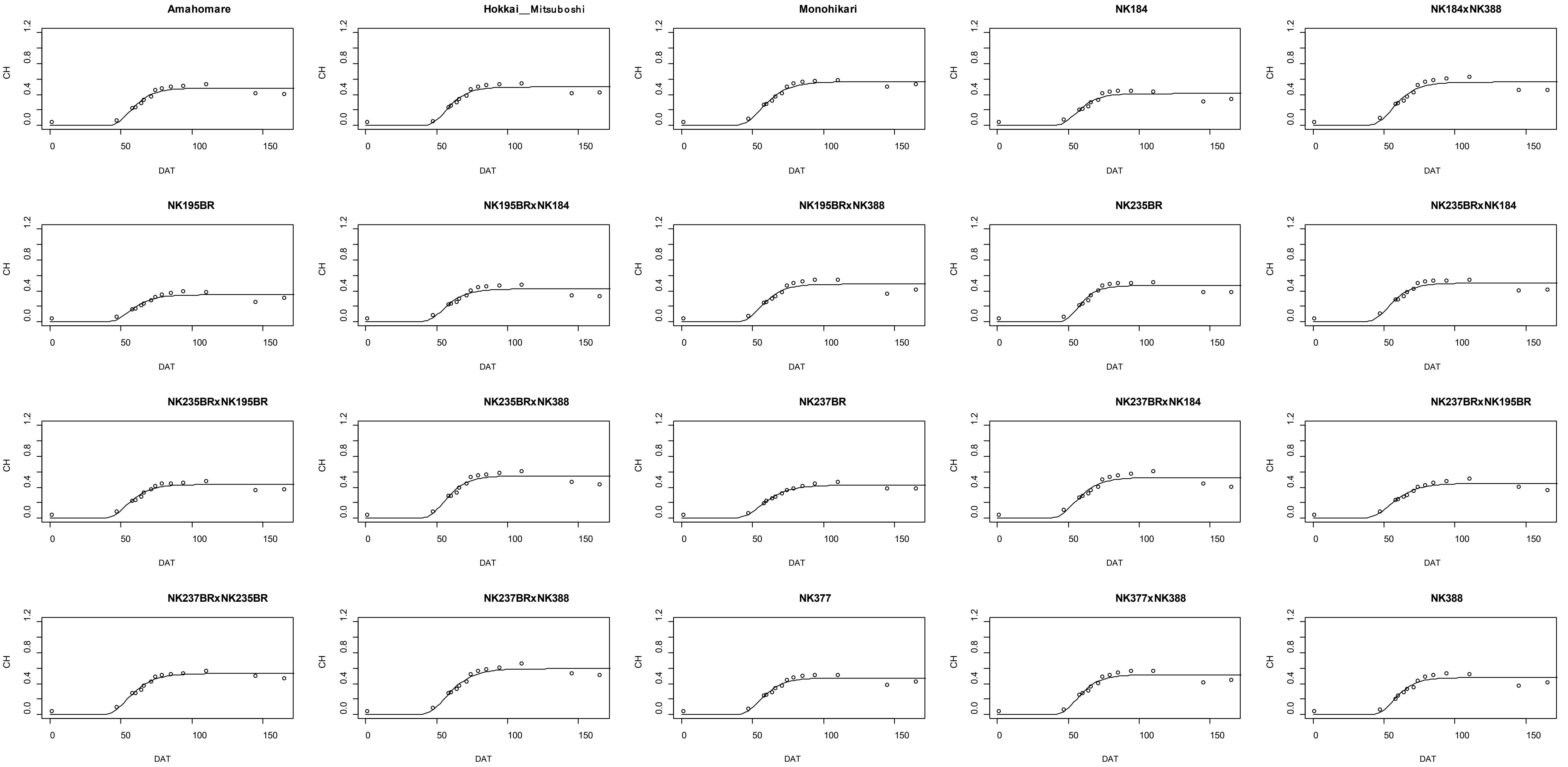
Amahomare
Hokkai__Mitsuboshi
Monohikari
NK184
NK184xNK388
NK195BR
NK195BRxNK184
NK195BRxNK388
NK235BR
NK235BRxNK184
NK235BRxNK195BR
NK235BRxNK388
NK237BR
NK237BRxNK184
NK237BRxNK195BR
NK237BRxNK235BR
NK237BRxNK388
NK377
NK377xNK388
NK388
CH
DAT
0.0
0.4
0.8
1.2
0
50
100
150

CH (2021)

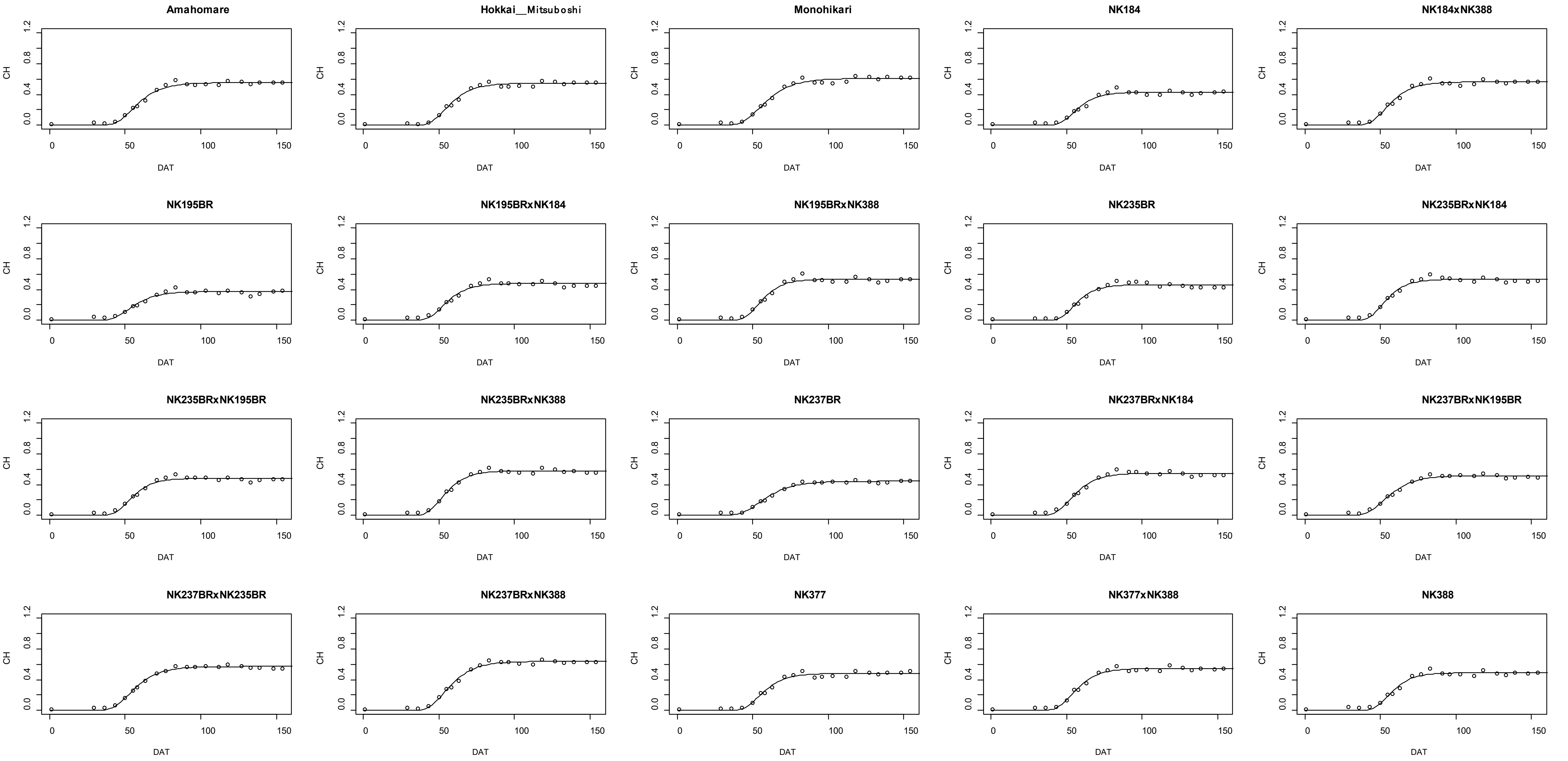

Amahomare
Hokkai__Mitsuboshi
Monohikari
NK184
NK184xNK388
NK195BR
NK195BRxNK184
NK195BRxNK388
NK235BR
NK235BRxNK184
NK235BRxNK195BR
NK235BRxNK388
NK237BR
NK237BRxNK184
NK237BRxNK195BR
NK237BRxNK235BR
NK237BRxNK388
NK377
NK377xNK388
NK388
CH
DAT
0.0
0.4
0.8
1.2
0
50
100
150
